# An expressed sequence tag (EST) library for *Drosophila serrata*, a model system for sexual selection and climatic adaptation studies

**DOI:** 10.1186/1471-2164-10-40

**Published:** 2009-01-21

**Authors:** Francesca D Frentiu, Marcin Adamski, Elizabeth A McGraw, Mark W Blows, Stephen F Chenoweth

**Affiliations:** 1 School of Biological Sciences, University of Queensland, St Lucia, QLD 4072, Australia; 2 Sars International Centre for Marine Molecular Biology, Bergen, Norway

## Abstract

**Background:**

The native Australian fly *Drosophila serrata *belongs to the highly speciose *montium *subgroup of the *melanogaster *species group. It has recently emerged as an excellent model system with which to address a number of important questions, including the evolution of traits under sexual selection and traits involved in climatic adaptation along latitudinal gradients. Understanding the molecular genetic basis of such traits has been limited by a lack of genomic resources for this species. Here, we present the first expressed sequence tag (EST) collection for *D. serrata *that will enable the identification of genes underlying sexually-selected phenotypes and physiological responses to environmental change and may help resolve controversial phylogenetic relationships within the *montium *subgroup.

**Results:**

A normalized cDNA library was constructed from whole fly bodies at several developmental stages, including larvae and adults. Assembly of 11,616 clones sequenced from the 3' end allowed us to identify 6,607 unique contigs, of which at least 90% encoded peptides. Partial transcripts were discovered from a variety of genes of evolutionary interest by BLASTing contigs against the 12 *Drosophila *genomes currently sequenced. By incorporating into the cDNA library multiple individuals from populations spanning a large portion of the geographical range of *D. serrata*, we were able to identify 11,057 putative single nucleotide polymorphisms (SNPs), with 278 different contigs having at least one "double hit" SNP that is highly likely to be a real polymorphism. At least 394 EST-associated microsatellite markers, representing 355 different contigs, were also found, providing an additional set of genetic markers. The assembled EST library is available online at .

**Conclusion:**

We have provided the first gene collection and largest set of polymorphic genetic markers, to date, for the fly *D. serrata*. The EST collection will provide much needed genomic resources for this model species and facilitate comparative evolutionary studies within the *montium *subgroup of the *D. melanogaster *lineage.

## Background

The genus *Drosophila *has proved to be one of the most useful groups of organisms with which to investigate fundamental questions in biology. The *melanogaster *group, in particular, has provided many of the model species currently studied by evolutionary biologists, including the native Australian fly *Drosophila serrata *[1]. *D. serrata *belongs to *montium*, the most speciose yet taxonomically least understood subgroup in the *melanogaster *group [2,3]. The utility of *D. serrata *in addressing evolutionary questions has been long recognized, for example in studies of speciation [4,5]. More recently, it has gained prominence as a model species for investigating the evolution of traits involved in sexual selection and mate recognition [6-8] and climatic adaptation [9-11]. The identification of functional genetic variants underlying phenotypes of interest, however, has been limited by the absence of a species-specific gene collection.

In recent years *Drosophila serrata *has become an important system with which to investigate sexual selection. The fly uses a blend of cuticular hydrocarbons (CHCs) as contact pheromones for mate and species recognition [6]. Studies utilizing *D. serrata *have, for example, investigated the evolution of sexual dimorphism in CHCs [12,13], divergence of mating preference in novel environments [14] and post-copulatory sexual selection [15]. Population differences in sexual selection regimes and the underlying genetic architecture of CHCs have also been identified [16,17], raising the possibility that different loci and/or alleles may be implicated in generating these phenotypes in each population. Although the quantitative genetic basis of CHCs under sexual selection is well understood in this species [18-20], the molecular genetic basis of these phenotypes remains unknown.

**Figure 1:**
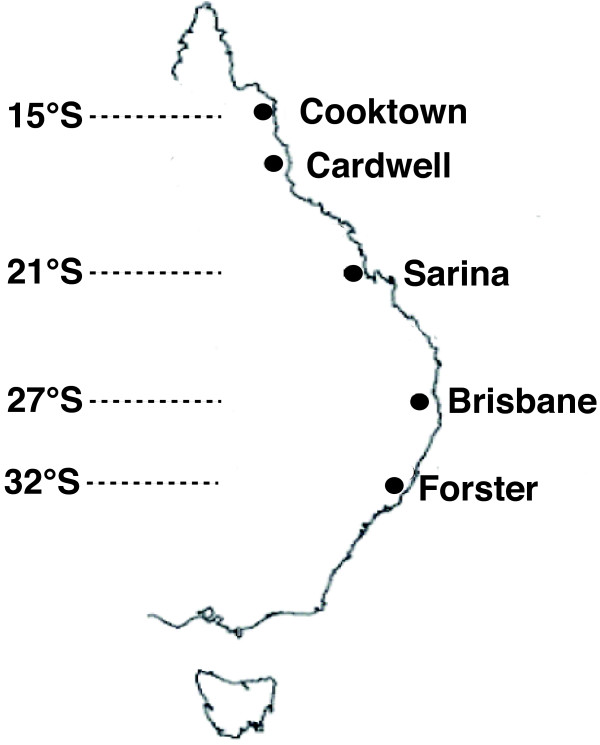
**Map of the east coast of Australia**. Populations of *D. serrata *sampled in this study are indicated by filled in circles.

*Drosophila serrata *has also proved very useful for understanding the process of climatic adaptation. Its distribution spans at least 17° in latitude (Figure 1) along a narrow band of suitable habitat on the east coast of Australia and populations experience a variety of temperatures and humidity gradients [21,22]. For example, latitudinal clines have been found in traits such as cold resistance [10,23], viability [10], developmental time [10], body size [9], wing shape [24] and CHC profile [8,16]. Chromosomal inversions are also known to vary in frequency along the latitudinal gradient [25] and may host co-evolving alleles at genes involved in climatic adaptation [26]. Mapping genes to particular inversions and identifying genomic regions involved in adaptation along the cline require a collection of molecular markers spanning the *D. serrata *genome.

The utility of *Drosophila serrata *to comparative studies (e.g. sexual character evolution) requires an accurate reconstruction of evolutionary relationships. The resolution of systematic relationships in the *melanogaster *group, however, has proved notoriously problematic [27-30]. Within the *montium *subgoup, in particular, the high degree of morphological convergence and similarity in male genitalia coupled with limited fossil representation has meant that taxonomic relationships are particularly unclear [3], necessitating a gene-based approach. Attempts to resolve phylogenetic relationships within the *montium *subgroup have so far been both sparse and based on very few genes [31], although relationships among the Australian species have recently received more attention [22,32,33]. Resolution of phylogenetic relationships in the highly speciose *montium *subgroup requires the development of additional genetic markers.

A cost-effective way of identifying a large number of genetic loci is to build an EST library [34], especially for species possessing a large genome and without a genome sequencing project. ESTs are single read sequences produced from sequencing an mRNA pool that samples the transcribed genes within a given set of tissues, individuals or populations. An EST library represents a resource that can be used for many downstream applications to address questions in evolution and ecology. For example, an EST collection can aid identification of genes underlying phenotypes of interest through the development of expression arrays [35] and provide a wealth of markers that may offer resolution of previously problematic phylogenetic relationships [36]. Additionally, single nucleotide polymorphisms (SNPs) and simple sequence repeat (SSR) markers (e.g. microsatellites) occurring within EST regions provide a source of potential markers for QTL mapping applications [37] and population genomic studies [38,39].

Here, we describe an EST collection from a normalized whole body library for *Drosophila serrata *as a genomic resource for this model species. The study was designed to simultaneously identify sets of genes potentially involved in the expression of traits of interest to evolutionary biologists and to provide an array of molecular markers for population genomic studies by incorporating multiple individuals from several natural populations of the species. We present the sequences of 6,607 putative genes and outline the discovery of numerous microsatellite and SNP markers present in the dataset. Using this EST collection, we have identified several genes which we hypothesize may be involved in the expression of traits that are implicated in sexual selection and climatic adaptation in *D. serrata*. Additionally, we have identified genetic loci that may eventually resolve the phylogeny of the *montium *subgroup. Individual EST reads are available from Genbank and dbEST (accessions FK858115 – FK867478 and 59290665 – 59300028 respectively) and from .

## Results

### EST Statistics

We sequenced a total of 11,616 ESTs from the 3' end (see Methods for explanation of sequencing rationale), of which 9,738 were of sufficiently high quality and length to process further. The EST sequences were assembled into a total of 6,607 contigs (note that we use Staden's 1980 original definition of a contig [40] which allows for single sequence contigs, here referred to as singleton contigs). Most contigs were represented by single sequences (5,419 out of 6607; Table 1), with an average length of 575 bp. The 1,188 contigs represented by at least two sequences were slightly longer on average than singletons (795 bp vs. 575 bp; Table 1). The majority of contigs (6,009 out of 6,607; Table 1) were found to be coding for peptides, suggesting that our strategy of sequencing from the 3' end was successful in identifying both protein coding sequences and 3' UTRs. Fewer than 10% of contigs represented sequences that included transposable elements (TE), pseudogenes, noncoding RNAs (ncRNA), microRNAs (miRNA) and transfer RNAs (tRNA) (Table 1).

**Table 1:** EST statistics

	Total number	Length (bases)		
		Min	Max	Average

Contigs (≥ 2 ESTs)	1188	113	1502	795
Contigs (singletons)	5419*	30	1054	575
Contigs – peptides	6009	60	1502	630
Contigs – other^#^	585	47	1411	467

The number and average length (bp) of contigs obtained from the *D. serrata *EST library.

* Includes singletons of high (CL0; N = 3639) and poor (CLx; N = 1780) quality. ^# ^Includes transposable elements, microRNAs (miRNA), noncoding RNAs (ncRNA), pseudogenes and transfer RNAs (tRNAs).

### EST annotation and identification of genes of interest

The 6,607 contigs were queried against all *Drosophila *protein coding sequences available in Genbank nr and all mRNA sequences in FlyBase at 01/03/2007 using BLAST [41]. All but 13 contigs returned hits to other *Drosophila *sequences, allowing us to assign putative functions to almost the entire EST library by using the top hit from the BLAST search. This approach has the caveat that sequence homology does not imply functional homology due to possible divergence of gene functions among species. At least 66% of the contigs had BLAST hits with e-values below 10E-05, suggesting significant sequence homology of our transcripts to genes from other *Drosophila *species. A number of partial transcripts were identified from genes implicated in CHC biosynthesis (e.g. fatty acid desaturases and elongases, [42]) and sexual selection in other *Drosophila *species (Table 2, [43-48]). Partial transcripts were also found from genes that may underlie traits involved in climatic adaptation in *Drosophila melanogaster*, for example heat shock proteins (Table 2, [49-55]. Additionally, a number of transcripts were identified tagging other genes of interest, for example in resolving phylogenetic relationships and rates of evolution in *montium *subgroup species (Table 2, [27,56,57]).

**Table 2:** EST contigs annotated to genes of interest

Gene	Contig ID	Length (bp)	Reference
	Sexual selection and CHCs		

Acp70A-1	CL0Est000004995273P08	157	[47]
*elongase *(CG16905-PA)	CL336Contig1	788	[44]
*desat1*	CL36Contig1	1222	[46]
*desat2*	CL1138Contig1	779	[46]
*Fad2 *CG7923-PA	CL67Contig1	774	[45]
Protein, ejaculatory bulb (CG2668-PA)	CL833Contig1	745	[48,80]
Sperm protein	CL448Contig1	718	-
Yolk protein 2 (CG2979-PA)	CL2Contig1	803	[43]
Yolk protein 3	CL9Contig1	848	[43]

	Climatic adaptation		

*Alcohol dehydrogenase *(Adh)	CL134Contig1	730	[55]
*Glycerol 3 phosphate dehydrogenase *(GPDH)	CL121Contig1	787	[51]
*Hsp23*	CL0EST000004965173F20	503	[54]
*Hsp26*	CL14Contig2	839	[49]
*Hsp83*	CL106Contig1	783	[52]
*Turandot *(*Tot*)	CL106Contig1	783	[50]
*Trehalase *(*Tre*)	CL753Contig1	727	[53]

	Phylogenetic		

*amylase*	CL158Contig1	899	[27]
*α-tubulin *(CG1913-PA)	CL110Contig1	768	[56]
*β-tubulin *(CG9277-PA)	CL37Contig1	795	[56]
Cytochrome oxidase subunit II (COII)	CL0Est000004981673F03	574	[27]
*Phosphoglucoisomerase *(PGI)	CL217Contig1	1010	[27]
Rhodopsin (CG5638-PA)	CL0Est000004965473E08	434	[57]

*D. serrata *contigs annotated to *Drosophila *genes of interest from sexual selection, climatic adaptation and phylogenetic studies. Contig identification codes are those used on the *D. serrata *web database .

**Figure 2:**
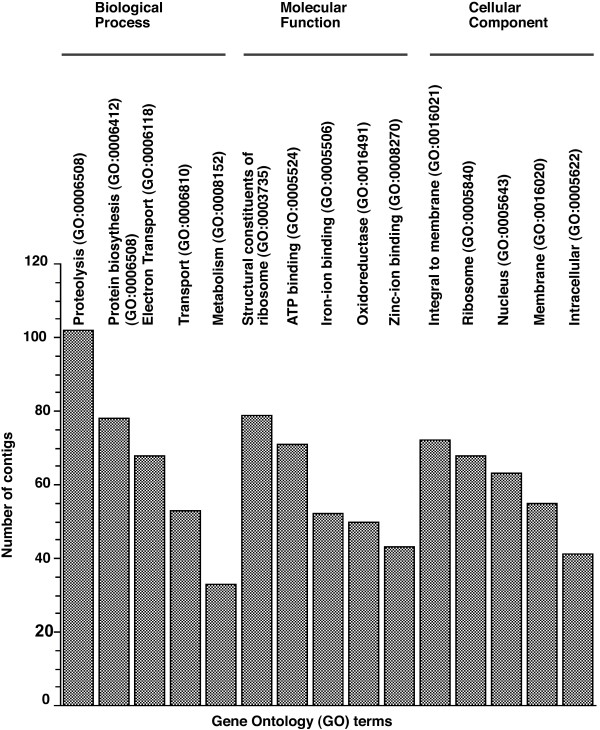
**Distribution of GO terms**. The five most frequently represented GO terms for each of three major gene functions in the *D. serrata *EST library, as indicated by the number of contigs in each category.

Despite the moderate number of ESTs sequenced, the library captured a range of types of transcripts, as indicated by the number of Gene Ontology (GO) [58] terms assigned to contigs. A total of 465 GO terms were assigned to contigs in our library, including 72 Cellular Component, 193 Biological Process and 200 Molecular Function. The five most commonly represented GO terms in our library were assigned to at least 30 contigs, with the largest category being proteolysis in Biological Process (Figure 2). The most frequently represented categories included housekeeping genes, for example structural components of ribosomes and protein biosynthesis genes (Figure 2).

The chromosomal distribution of ESTs surveyed in this library was largely similar to that present in *Drosophila melanogaster*. Chromosomes 2, 3, X and 4 accounted for approximately 37%, 44%, 16% and 0.01% respectively of the total percentage of ESTs discovered in *D. serrata*. This distribution is close to that observed for *D. melanogaster*, with chromosomes 2, 3, X and 4 accounting for 37%, 44%, 18% and ~0.01% respectively of the total genome [59]. X-linked sequences are slightly underrepresented in *D. serrata *compared to *D. melanogaster*, a result that may be due to the small number of transcripts surveyed in our study or may reflect real biological differences between the species. The number of putative genes identified in our library that were protein coding was 6,009, resulting in a gene density of ~5 genes per Kb if we assume the *D. melanogaster *euchromatin amount [59]. Given our limited sequencing effort necessitated by cost constraints, these numbers compare well given the estimate of ~14,000 genes in *D. melanogster *[59,60] and suggest we have captured slightly less than half of the genes present in the genome. However, a large number were loci that are identified as CG-id only on Flybase, of which on average over half are estimated to be computational predictions with uncharacterized functions and may not represent real genes [60].

### Genetic markers for mapping and population studies

We identified numerous microsatellite and single nucleotide polymorphism (SNP) markers from our EST library using the software SciRoKo version 3.1 [61]. To maximize the chance of a potential microsatellite marker being polymorphic we restricted our search for di-, tri-, tetra-, penta- and hexa- nucleotide motifs that were present repeated at least six times in the contig sequences. If only perfect repeat microsatellites are considered we found a total of 394 markers comprising 295 di-, 95 tri-, 3 tetra- and 1 hexanucleotide repeats (Table 3), representing 355 different contigs. The majority (83%) was found in protein coding transcripts and the rest were found in transposable elements. When repeats were allowed to have a conservative degree of mismatch (≤ 2 bp), at least twice as many microsatellite markers were revealed (Table 3), representing 836 independent contigs. Again, the majority of imperfect microsatellite markers (85%) were found in protein coding sequences, with the rest being found in transposable elements.

**Table 3:** EST-derived microsatellite statistics

	Perfect repeats		Imperfect repeats (< 2 mismatches)	
Repeat size	Number	Average length (± S. E.)	Number	Average length (± S. E.)

2	295	18.87 (± 6.43)	356	23.31 (± 9.22)
3	95	20.58 (± 3.40)	292	22.43 (± 11.61)
4	3	29.00 (± 4.97)	116	19.81 (± 8.42)
5	-	-	129	17.99 (± 4.08)
6	1	44.00 (± 0)	123	23.98 (± 8.31)

The number and average length of microsatellites (SSRs) found in the *D. serrata *EST library, based on 6,607 contigs. The minimum-repeat number for both analyses was six.

**Figure 3:**
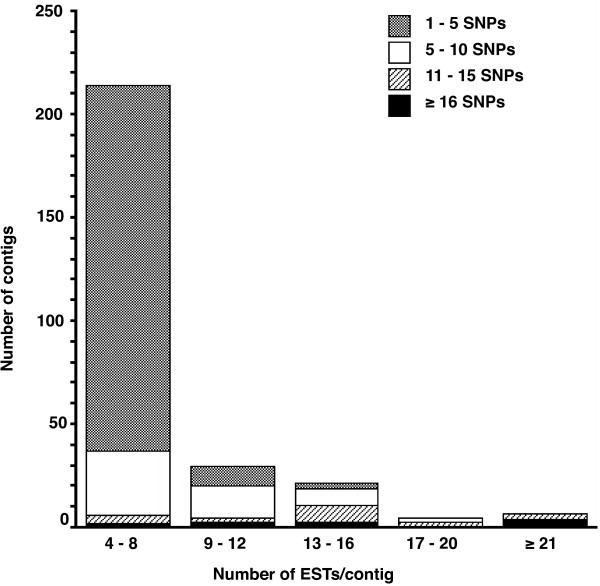
**SNP discovery versus alignment depth**. Proportion of SNPs identified in the *D. serrata *EST database versus contig alignment depth, restricted to the dataset of 1,254 'double hit' high quality SNPs. The total number of contigs for each alignment depth class (number of ESTs/contig) is represented by the height of the column. The different shading patterns within a column indicate the number of SNP classes (e.g. 1–5 SNPs/contig). Most contigs were shallow in depth (4–8 ESTs/contig) and contained relatively few (1–5 SNPs) sequence variants per contig. Note that here alignment depth is not constant across all sites along the contig and instead denotes the total number of ESTs per contig.

We found a total of 11,057 putative SNP markers in our EST collection. Close to a third of these SNPs (3,219) were identified from contigs that comprised only two ESTs. Although a large proportion of these are likely to be real polymorphisms, in practice it is difficult to identify *a priori *without further testing which of these 3,219 SNPs are also spurious mutations introduced during reverse transcription, cDNA library construction and contig assembly. A more reliable way of identifying real polymorphisms is to only consider SNPs found in at least two ESTs from contigs comprising at least four sequences [62]. A total of 5,866 SNPs were found in contigs of at least four ESTs, but of these only 1,438 were represented by at least two sequences. A large proportion (1,254) of these 'double-hit' SNPs [63] were also of high quality, with at least one of the base variants having a mean PHRED quality score of ≥ 20. The 'double-hit SNPs' occurred in 278 individual contigs. Most SNPs were discovered in contigs with relatively low sequence coverage (Figure 3) and many contigs displaying variation harboured between 1 and 5 SNPs (class 4–8 ESTs/contig; Figure 3). Although increasing alignment depth (i.e. the number of ESTs/contig) resulted in increased SNP discovery, the actual number of contigs harbouring very many SNPs was low (Figure 3). Our strategy of incorporating multiple individuals in the cDNA pool used to produce the EST library has resulted in a large number of potential genetic markers for *D. serrata *to be used in mapping and population genomic studies.

### Drosophila serrata EST web database

The *D. serrata *EST collection presented here is available on the web at . The site comprises several pages from which EST contigs are available for download in several formats (e.g. FASTA). The 'Search' page allows the user to find particular transcripts either by contig identity, annotation or chromosome location (based on *Drosophila melanogaster *chromosome designation) and for particular microsatellites by sequence type and repeat number. It should be noted that the chromosome arm labeled as 3R of *D. serrata *by [25,64] is equivalent to the 2L and not the 3R of *D. melanogaster *due to an earlier labeling error [64]. Workers wishing to map genes to the *D. serrata *chromosome labeled as 3R by [25,64] using sequence homology with *D. melanogaster *should choose genes found on the 2L arm of the latter species. Contigs can also be viewed either by gene name or by GO terminology. A TAB formatted file provides additional information for each contig, including contig length and results of BLAST searches against other databases (see above). Detailed BLAST results are also available as XML files linked to each contig. The *D. serrata *EST web database can also be queried using the BLAST tool hosted on the site. Contig identification codes fall into three categories: 1) contigs comprising at least two transcripts, denoted by CL followed by a number from 1 to 1,210; 2) singleton contigs of good sequence quality, denoted by CL0; and 3) singleton contigs of poorer sequence quality, denoted by CLx. A total of 9,364 individual EST reads, excluding rRNAs, RNAs of mitochondrial origin and sequences shorter than 50 bp, have been deposited with Genbank (accessions FK858115 – FK867478) and dbEST (accessions 59290665 – 59300028).

## Discussion

Here we have described an EST collection for the native Australian fly *Drosophila serrata *that has become a prominent model for studies of sexual selection and climatic adaptation. Using a normalized library from whole fly bodies and 3' end Sanger sequencing of clones, we generated a functionally diverse collection of 6,607 EST contigs, the majority of which encoded peptides in addition to 3' UTRs. Using BLAST analyses we were able to successfully assign putative functions to EST contigs according to sequence homology with the 12 *Drosophila *genomes available in FlyBase . This unique collection of ESTs will greatly facilitate the development of genomic applications in *D. serrata*, such as gene expression arrays.

Our approach of incorporating multiple individuals from collections spanning the geographic distribution of the species allowed us to generate genetic markers for population level studies. We identified at least 394 EST-associated microsatellites and, by aligning in a single contig sequences representing different naturally occurring alleles, we discovered at least 1,254 high quality SNP markers. We have already designed and tested primers for 150 EST-derived microsatellites currently being used in QTL mapping studies of traits under sexual selection in *D. serrata*. Although the total number of SNPs found in the EST collection was 11,210, over half of these were found in contigs of sequence depth ≤ 3 sequences. An additional proportion was found in contigs of depth of at least four sequences but represented by only one allele. An unknown fraction of these SNPs is probably due to reverse transcription and amplification errors during library synthesis, poor quality sequence and assembly and alignment errors rather than genuine polymorphisms. Despite this, we were still able to identify a large number of potentially polymorphic SNPs by considering only those found in contigs of at least four sequences and where the minor allele is found in two transcripts or more. Our results and those of previous workers [63] suggest that SNP identification can be highly successful from normalized EST libraries if multiple individuals are included in the original mRNA pool, although polymorphic marker identification may be biased towards those found in highly transcribed genes.

Gene annotation against the 12 *Drosophila *genomes available in FlyBase also allowed identification of genes that may underlie traits of evolutionary and ecological interest. We found at least nine genes that may be involved in the expression of traits under sexual selection (Table 2) such as cuticular hydrocarbons. For example, we have identified partial transcripts from two desaturases (*desat1 *and *desat2*) and an elongase involved in CHC biosythesis [42] (Table 2). Functional polymorphism in desaturase and elongase genes has been shown to produce different CHC profiles in *Drosophila simulans *and *D. melanogaster *[44,46,65]. The partial transcripts of the desaturase and elongase genes have already been used to design primers for rapid amplification of cDNA ends (RACE) studies, with the goal of obtaining full-length sequences. We have also identified an accessory protein (Acp70A-1; Table 2) transcript in *D. serrata *that may be involved in male sperm and seminal fluid traits, as has been found in *D. melanogaster *[47,66]. Previous work has shown that *D. serrata *exhibits particularly high female remating rates and levels of multiple paternity in both lab and field populations [15]. Insemination rates and multiple paternity levels may be highly dependent on sperm characteristics and may reflect genetic polymorphism among males at particular genes [47,67].

Our EST collection and array of microsatellite and SNP makers may also facilitate further exploration of the molecular genetic basis of climatic adaptation along a latitudinal cline in Australian *Drosophila serrata*. First, we found a number of heat shock protein (*Hsp*) and other genes (Table 2) that are thought to modulate physiological tolerance to temperature in *D. melanogaster *[68,69]. One of these genes, *Hsp83 *(also known as *Hsp90*) has been implicated in cold resistance in the closely related species *D. birchii *[70]. Second, genes involved in CHC biosynthesis may also be involved in desiccation resistance along the cline since cuticular hydrocarbons also serve to waterproof the insect [42]. The genes *desat1*, *desat2 *and *elongase *are of particular interest since they have been shown to mediate CHC polymorphism in *D. melanogaster *[44-46] a trait that displays clinal variation in Australia in both *Drosophila *species [21]. For example, *desat2 *is involved in the biosynthesis of 5,9 dienes in *D. melanogaster *[46], compounds which are also found in *D. serrata *[71]. The gene *elongase *is also involved in the synthesis of longer chained dienes in *D. melanogaster *[44] that are also expressed in *D. serrata*. Third, the SNPs and microsatellites found in the EST collection could be used to identify genomic regions that may be involved in climatic adaptation. Pronounced genetic divergence at genes underlying phenotypes under selection compared to neutrally evolving markers and specific patterns of sequence polymorphism may indicate areas of the genome involved in adaptation along a cline [72]. Fourth, microarray probes can now be designed to detect changes in gene regulation in response to selection on particular phenotypic traits known to vary clinally. Fifth, our EST collection facilitates physical mapping of particular genes to chromosomal inversions in *D. serrata *and may help resolve the degree of chromosomal synteny between *montium *and other subgroups of the *D. melanogaster *group.

Finally, we were able to identify genes that should be of phylogenetic utility in resolving relationships within the *montium *subgroup of the *Drosophila melanogaster *group, which to date remain unclear [31]. EST collections provide a means of obtaining partial sequence for many genes at once, providing potential resolution of previously problematic taxonomic relationships [36]. Partial transcripts from several genes of phylogenetic interest (e.g. *aconitase*, *PGI*) were found in our EST collection and potentially more might be identified by BLAST searches using other phylogenetically relevant genes as queries against our database. At the time of development and analysis of our *D. serrata *EST database, *D. kikkawai, D. jambulina *and *D. birchii *were the only other *montium *species with any significant molecular data in Genbank, out of a subgroup of over 100 species. Phylogenetic markers may be developed using data from these four species for use in other *montium *taxa, by designing degenerate primers for example. Further work will help resolve phylogenetic relationships in this important subgroup and will allow accurate tracing of the evolutionary history of interesting traits, among other questions.

## Conclusion

*Drosophila serrata *is a native Australian fly that has recently become a prominent model system with which to investigate the evolution of traits under sexual selection and traits involved in climatic adaptation. Understanding the molecular genetic basis of traits of interest to evolutionary biologists has been hampered by a lack of genomic resources for this species. Here, we have reported the development of an EST library for *D. serrata *from whole fly bodies at several stages of development. We sequenced 11,616 EST clones from the 3' end that were assembled into 6,607 contigs. The majority of contigs was found to contain peptide-coding sequence in addition to 3' UTR and represented a substantially diverse set of gene functions. At least 394 potentially polymorphic microsatellites were found associated with the EST contigs. By incorporating multiple individuals from five populations throughout the distribution of *D. serrata*, we were able to identify a large number of SNPs, including at least 1,254 'double hit', high quality sequence variants. The EST library contained partial transcripts from genes of interest to studies investigating the molecular genetic basis of sexual sexually selected traits, for example desaturases involved in CHC biosynthesis. A number of genes were also discovered that may code for phenotypes implicated in climatic adaptation along a latitudinal gradient in *D. serrata*, for example heat shock proteins. The EST library has also revealed a number of genes of potential phylogenetic utility and that may help resolve evolutionary relationships within the highly speciose *montium *subgroup. We anticipate the genomic resources provided by the EST library will facilitate numerous downstream applications that will answer fundamental questions in evolutionary biology using *D. serrata *as a model organism.

## Methods

### Fly populations and RNA isolation

Our EST project was designed to simultaneously generate a library of putative genes for sexually selected traits and polymorphic markers for population level studies. The *D. serrata *sample comprised larvae at the last instar (N = 30) and five adult stages: day 0 (emergence) (30 females, 30 males), day 1 (25 females, 30 males), day 2 (22 females, 28 males), day 3 (30 females, 28 males) and day 4 (23 females, 24 males). Several life stages were used in order to maximize gene discovery for further microarray and gene expression studies. Within the sample used, five populations were represented, spanning the geographical distribution of *D. serrata *on the east coast of Australia: Cooktown, Cardwell, Sarina, Brisbane and Forster (Figure 1). Flies were obtained from mass bred cultures established from wild-caught inseminated females (N = 20) from each of the five geographical locations and maintained at large population sizes in the laboratory for approximately two years.

Individuals were removed from rearing bottles and immediately frozen in liquid nitrogen. Total RNA was extracted from whole fly bodies using Trizol (Invitrogen, Australia) and mRNA purified using the GenElute mRNA miniprep kit (Sigma-Aldrich, Australia). Equimolar amounts of mRNA from each sex and each life stage were pooled to construct a single cDNA library. Library construction and normalization were performed by Agencourt Biosciences (MA, USA) according to proprietary protocols.

### EST sequencing and assembly

Sequencing was performed from the 3' end of transcripts. There are several advantages to this strategy. First, identification of unique contigs (and therefore putatively unique genes) is more reliable than in 5' sequencing projects since alternative splicing is much more frequent in 5' as opposed to 3' UTR [73], meaning that it is more likely for transcripts of the same gene to share a common polyA tail. This reliability combined with the depth of alignments containing transcripts from multiple individuals facilitates discovery of polymorphic molecular markers, like SNPs and microsatellites [63]. Second, they represent much better features for expression arrays since cross-hybridization amongst gene families is reduced due to 3' UTRs being generally less conserved than coding regions.

Sanger sequencing of the 3' ends of clones was performed by Agencourt Biosciences (MA, USA), using a proprietary sequencing primer. ESTs were clustered using the TGICL tool  under the default parameters, with vector sequences and polyA tails masked. ESTs which were not assembled into any contig were identified and the quality of their sequence was determined using the program LUCY2 [74] with the following parameters: error 0.025 – 0.02, bracket 20 – 0.02 and window (20 0.01 10 0.03). The shortest accepted length of good quality sequence was 18 bp. Single ESTs that passed quality trimming were then grouped into an artificial cluster denoted CL0 and ESTs that did not pass were grouped into another artificial cluster denoted CLx. Therefore, sequences in the cluster CL0 were quality trimmed whereas sequences in the cluster CLx were not. Assembled contigs and individual ESTs from clusters CL0 and CLx were then annotated in the same way.

### Annotation of genes via sequence homology with other Drosophila

The 6,607 sequences (contigs and unassembled ESTs) were first queried using nucleotide versus protein blastx against the NCBI nr (non-redundant) protein database, limited to *Drosophila *entries. Blastx parameters were set to: amino acid substitution matrix BLOSUM-62 [75], a statistical significance threshold of 10 for database matches [76] and costs to open an alignment gap and extend a gap of 11 and 1 respectively. Query sequences were filtered for low compositional complexity using the program SEG [77]. Sequences that did not match any proteins were annotated using the following *Drosophila melanogaster *release R5.1 sequences from FlyBase : microRNAs, miscellaneous RNAs, noncoding RNAs, all pseudogenes, all transposons and all transfer RNAs. Searches against FlyBase were performed using nucleotide vs. nucleotide blastn. Output from the blastx search was functionally annotated with Gene Ontology (GO) terminology using the blast2go tool with the default parameters [78]. Genomic localization of the ESTs was done using the tool Exonerate [79] and the *D. melanogaster *genome as reference.

### Microsatellite and SNP marker identification

The database of 6,607 contigs was mined for microsatellite and SNP markers to be used in future population genetic studies. For the identification of microsatellite markers, we used the program SciRoKo version 3.1 [61] that can easily identify di- to hexanucleotide repeats. Searches were conducted to identify both uninterrupted and interrupted (≤ 2 bp mismatch) motifs, with a minimum number of repeat units of six.

Putative SNP markers were identified from all contigs with at least two ESTs by using a custom Perl script. Polymorphic sites were denoted using an IUB code (e.g. Y). Each SNP was assigned a quality score that was an average of individual PHRED scores for each sequence at that base position. ESTs often contain error mutations introduced during the reverse transcription process and spurious polymorphism may arise in contigs from incorrect assembly. Consequently, using the rationale of [62], we also identified SNPs that are represented by at least two sequences in contigs with at least four ESTs.

## Competing interests

The authors declare that they have no competing interests.

## Authors' contributions

FDF wrote the manuscript and performed analyses on the EST dataset. MA ran the bioinformatic pipeline used to assemble ESTs, wrote Perl scripts to identify SNPs and created the EST website. SFC, MWB and EAM conceived, designed and coordinated the study. All authors read, commented and approved the final manuscript.
